# Exploring Pediatric Resident Attitudes and Preferences for Board Exam Preparation

**DOI:** 10.7759/cureus.8022

**Published:** 2020-05-08

**Authors:** Alex Liu, Suzanne Reed, John D Mahan, Rebecca Wallihan

**Affiliations:** 1 Pediatrics, The Ohio State University College of Medicine, Columbus, USA; 2 Pediatric Oncology, The Ohio State University, Nationwide Children's Hospital, Columbus, USA; 3 Pediatric Nephrology, The Ohio State University, Nationwide Children's Hospital, Columbus, USA; 4 Pediatric Infectious Diseases, Nationwide Children's Hospital, Columbus, USA

**Keywords:** medical education, board exam, attitudes, graduate medical education

## Abstract

Objective

The American Board of Pediatrics Certifying Exam (ABP CE) is a high stakes exam and is important for employment and fellowship opportunities in pediatrics. Although research has suggested interventions which may improve scores, little research has focused on resident perception of preparation for the ABP CE. In this study, we aimed to better define pediatric residents’ attitudes and preferences regarding preparation for the ABP CE.

Methods

Pediatric residents from one residency program were invited to participate in focus groups to discuss their attitudes and preferences on board exam preparation for the ABP CE. Focus groups were audio recorded and transcribed. Conventional content analysis was used to analyze qualitative data. From the transcripts, authors developed codes through an iterative process, which were then organized into categories. These categories were then grouped into themes.

Results

Nineteen residents participated in three focus groups. Focus group transcription analysis resulted in 49 codes, which were sorted into 26 categories. The categories were then grouped into four key themes: 1) the ABP CE is not immediately important early in residency; 2) more personalized guidance is preferred; 3) consistent board preparation focus from the residency program is valued; 4) learning format is important.

Conclusions

Residents believe preparation for the ABP CE increases in importance as they progress through residency, and they desire more personalized, consistent, and structured focus from their training program related to ABP CE preparation. Attention to these perceptions can help guide pediatric residency program leadership in developing effective board exam preparation strategies and curricula for their residents.

## Introduction

The American Board of Pediatrics Certifying Exam (ABP CE) is required for pediatricians to become board certified [1]. Although voluntary, achieving certification is important to most trainees and may be required for employment opportunities after residency. Individuals lose their ABP eligibility status seven years after graduation if they have not passed the ABP CE, and a retraining program is required to regain board eligibility, making the ABP CE a high-stakes exam [2].

To improve performance on the ABP CE, pediatric residency programs typically implement a variety of educational interventions to prepare trainees for this exam. Research on the efficacy of various board review methods has been conducted in different specialties [3-6]. However, there are little data that examine resident attitudes and preferences for board preparation. A study of anesthesiology residents used surveys to capture learner preferences and how these preferences correlated to their In-Training Exams [7]. In others studies, there has been some exploration of factors leading to acceptance of board preparation programming and learner preferences, but this was not the primary aim of these studies [8-10]. These studies did not focus on understanding overarching attitudes and preferences of the residents, but rather focused only on their perceptions related to a specific educational platform.

Understanding learning styles and preferences is an important part of curriculum design and development. In the commonly utilized Kern’s approach to curriculum development, understanding learner preferences is a key component of a targeted needs assessment and informs the development of appropriate educational strategies [11]. Additionally, millennial learners - who are becoming the most prevalent generation of medical learners - often have different preferences and expectations than previous generations of learners [12, 13]. In the present study, we sought to better understand the preferences of pediatric residents for and attitudes towards ABP examination preparation, in effort to better inform curriculum design for board preparation education.

## Materials and methods

Research design

For this exploratory study, we used focus group-based qualitative research methodology to characterize pediatric resident attitudes and preferences for ABP exam preparation in a single institution in June 2018 [14]. Our program is part of a large, academic, free-standing children’s hospital in the Midwest, with approximately 120 total categorical pediatric residents at any one time. We chose this approach to more thoroughly explore and understand personal perspectives of the residents, in order to gain insight into their thoughts and attitudes. This study was submitted to the Nationwide Children’s Hospital Institutional Review Board and was deemed exempt.

Study setting and population

Subjects included in this study were categorical pediatric residents at Nationwide Children’s Hospital. We invited individuals from all training years (PL1 - PL3) to participate in three separate focus groups with the goal of 6-8 participants per group.

Focus group development and administration

Focus groups were designed according to best practices, with the goal of eliciting discussion and feedback about the pediatric board exam and board preparation in general [14]. Focus group participants were separated into three distinct groups based on training year to elucidate differences between the groups. Each focus group was moderated by at least two of the study investigators, depending on availability, with one as the moderator and one as the observer. Key questions included 1) “What are your feelings about board preparation, 2) “How do you think board exam preparation can be best integrated into residency training,” and 3) “How do you best prepare for board exams?” Key questions were developed based on the specific research question of this study. The moderator focused on facilitating discussion among participants, rather than engaging in interview-style question and answer. The key questions were used with each group but individuals were free to add additional insights and reflect and respond to comments of others in the group. Introductory, transition, and ending questions slightly varied depending on the conversation among each individual focus group [14]. Focus group discussions were recorded and transcribed manually without any identifying information.

Data analysis

We analyzed qualitative data from focus groups transcripts using conventional content analysis [15]. Investigators initially coded the focus group transcripts separately, then met to gain coding consensus. Transcripts were then re-coded according to the agreed upon codes. Codes were organized into concepts, which were then used to create overarching themes.

## Results

A total of three focus groups were conducted, one for each training year: Eight PL-1 residents, six PL-2 residents, and five PL-3 residents. Demographics of participating residents are found in Table 1.

**Table 1:** Focus Group Participant Demographics

Training Year	Total Participants	Male	Female
PL – 1	8	1	7
Pl – 2	6	0	6
Pl – 3	5	2	3
Total	19	3	10

**Figure 1:**
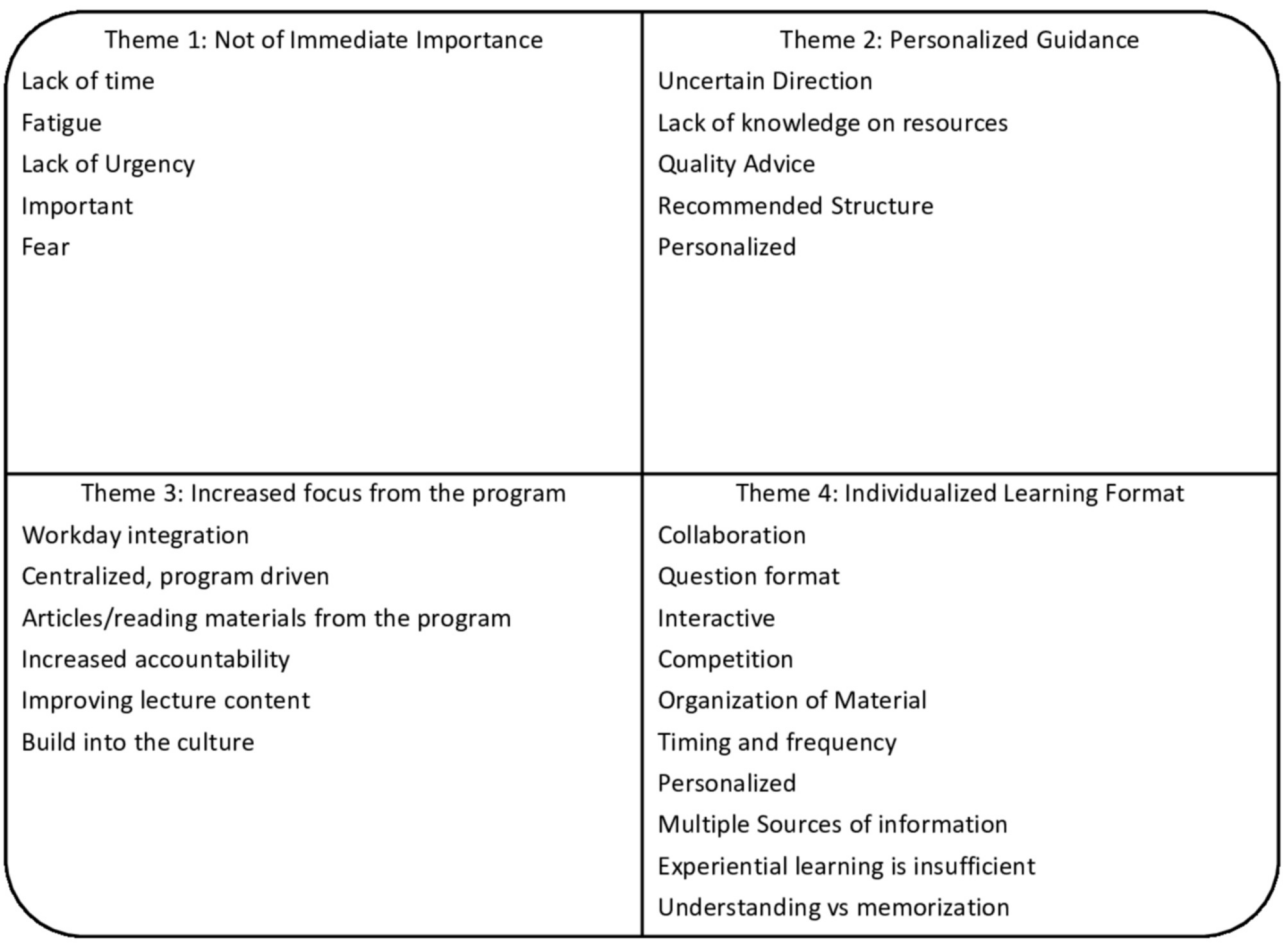
Categories and Themes Regarding Board Preparation from the Pediatric Residents

Focus group duration ranged from 45 to 60 minutes. Within the focus groups, several specific codes emerged across groups. The focus groups yielded 49 codes which were sorted into 26 categories. The categories yielded four themes regarding ABP CE preparation: 1) preparation for the ABP CE is not of immediate importance, particularly early in training; 2) more personalized guidance for board preparation is preferred; 3) increased board preparation emphasis from the residency program is valued; and 4) varied learning formats are key to individualized board preparation. Categories and themes are shown in Figure 1. Representative quotations within each theme are shown in Table 2.

**Table 2:** Representative Quotations from Major Themes

Theme 1: Not immediately important: “I guess the question is when to start, right? So the moment you start residency, you feel as if your learning has started, so being with patients is starting, but the boards questions should start in most lectures. … then all third years are like I wish I started sooner, all second years are like I should start halfway through the year, so really when is the question of starting and what are we doing to start?” – PL 1 “It just seems like there's other milestones ahead as well, that sort of make it seem further off that it probably really is. “– PL 2 “It’s something that is hard to do when the pressure of the test is not close. Like it feels like even if you try to study, it's not efficient studying.” – PL 3
Theme 2: Personalized guidance: “I guess I think of resources. There’s so much out there, a little overwhelming, but I think it is individualized, so giving an option of different ones, but nothing like, one required because, since people are learning differently.” – PL 1 “So I think that it would just make people feel calm going into this year if we felt that we had a really good session at that retreat where it was like, these are your options, this is what people like, this is what people haven't liked, you know, this is our plan for this year to get you ready” – PL 2 "Like I remember back in med school… here are all the options out there, here's my learning style, this is why it worked for me and that was really helpful when I was choosing what I was going do for board prep in medical school…we would really benefit from hearing from them what worked [for those who have passed boards].” – PL 3
Theme 3: Increased focus from the program: “Like I think its fine to like make our ILPs but then, we have to be personally responsible, obviously, but if we care as a program to do something, I think that part of it should be, ok my semi-annual review should be asking me the next time, hey have you read your peds in review articles this month and if not, how do we like get you to do that a little better.” – PL 1 “I think that having a dedicated time during like the work week hours is really the best way to do it because I think that everyone has good intentions if you try to do something like in the evening but I don't think that, you know, will actually work out.” – PL 2 “One program did it where they picked like June is when they would learn about, like GI, and so they get emailed out like 5 questions, like peds in review articles that relate to it and then like…some of them would also get like… divided out into board groups, kind of like a journal club or something. And then like, it's very like, this is what we do together in the program” – PL 3
Theme 4: Learning format: “I think it started as like one, videos, and like reading and annotations to understand the knowledge, right? So I think that's what we should be doing, like thinking about, if you are that type of learner, thinking about like understanding now because to do questions, you need to understand what the question's asking, and the concepts, and then, like we are all saying, like do questions to sort of focus in and apply the knowledge.” – PL 1 “I think just trickling a little bit consistently would be good. Even like I was thinking with our weekly updates, which the one document that I think everyone actually reads. We could put something on there, just like a board review question of the week.” – Pl 2 “the primary care lectures series, I can't stand because we come to this room and we sit here and just watch PowerPoint and wouldn't it be so much better to sit and do board questions in a small group together, cases, or something that is more interactive cause … like why not use that lecture series to do something more interactive” – PL 3

Theme 1: Boards are not of immediate importance

Residents emphasized that they found the ABP CE, and preparation for the ABP CE important, with some individuals expressing anxiety about the exam. However, residents found the exam was difficult to study for, citing that the exam was too far in the future and for many residents, motivation to study was hard to find until closer to the exam. Residents reported that the demands of residency also made it difficult to immediately prioritize the exam versus immediate obligations to the residency program. There was also a sense that without the program making it a priority, it was easy for the residents to view preparation as not important enough to invest time and energy at this stage.

Theme 2: Personalized guidance for board preparation is desired

Participants reported a lack of clear direction on how to best prepare for the ABP CE and that this was a barrier to engaging in preparation. They reported uncertainty in how to best approach board preparation and were unsure when it was necessary to start. Residents also reported uncertainty about the types and quality of board preparation resources. Participants desired identification and endorsement of reliable and vetted resources from the residency program, with third year residents stating there was often conflicting advice from others on how best to prepare. First year residents reported they would prefer more structure, such as having a centralized list of resources or a menu of recommended approaches. Participants also stressed that board preparation was very personal, with learners likely to have very different ideas on board preparation schedules, methods and motivation, arguing that individualized guidance would be most preferred and effective.

Theme 3: Increased focus on board preparation from the program is valued

Residents wanted the residency program to spend more time and resources on board preparation, and felt that this would spur resident motivation and engagement. In addition, residents reported that their work schedules often left them feeling as if they lacked enough time or were too tired to study for the ABP CE during training. They favored the residency program promoting a more active and apparent board preparation culture. Integration of board exam preparation into the workday was very important to them, with suggestions including working board practice questions into regular journal clubs and lectures, as well as stand-alone board preparation sessions. Some residents suggested that the residency program provide articles and reading materials. All groups stated that the residency program should hold the residents more accountable, with suggestions like individual board exam preparation tracking throughout training.

Theme 4: Varied learning formats for board preparation are important

Participants agreed that they had varied preferences regarding board preparation, and as such, having varied learning formats would be important for any group of residents. They strongly endorsed having a number of effective sources of board preparation information, and they emphasized the personalized nature of board preparation for individuals and how that aligned with serving different individual learning styles and methods. Different residents preferred using questions, videos, podcasts, and/or reading as effective methods to prepare for board exams. The timing and frequency of preparation were important factors to residents, with participants suggesting board preparation should be a consistent process and ideally started early in training. Residents preferred that material should be organized in blocks, with clear delineations between topics. In addition, residents also desired interactive learning, with residents suggesting the value of collaborative and competitive initiatives such as preparation in groups and competitions between various resident clinics. Participants commented that they viewed experiential learning during residency as important to their training but likely insufficient for board preparation. Participants felt that preparing for boards required efforts at both understanding and memorization.

## Discussion

In our study, we found four key themes related to pediatric resident attitudes towards ABP CE preparation: 1) preparation for the ABP CE is not of immediate importance early in residency; 2) more personalized guidance for board preparation is preferred; 3) increased board preparation emphasis from the residency program is valued; and 4) the learning format should be individualized for trainees.

We found that although residents considered the ABP CE an important exam, it was difficult for them to find motivation to study for the exam during residency, particularly in the midst of the demands of clinical training. This finding has not been emphasized in other literature but it does indicate a key perspective and role for program leadership in this regard. Although residents in other fields have expressed that their certification exams were important to them, difficulty in motivation to study during residency has not been reported [16]. Our residents reported factors such as fatigue and lack of time to prepare as contributing factors, which is not surprising given the rigor of residency training. Pediatric residents, in addition to managing their clinical work, have broad responsibilities ranging from teaching medical students, individual career preparation, personal academic and other projects, and fellowship/job applications, leaving residents feeling they have little time to devote to studying for the ABP CE. This reality argues for residency programs to consider developing methods to integrate dedicated time for board preparation into the curricula and specific efforts to emphasize the importance of early and consistent preparation for the ABP CE.

Focus group participants also reported a desire for more personalized guidance regarding the ABP CE. Residents described considerable uncertainty on how to approach preparing for the exam and that quality advice was important to them. This mirrors the results of a study on emergency medicine residents and their preferences for studying for their in-training exams. In this study, emergency medicine residents reported that they particularly valued peer or fellow resident recommendations for preparation [17]. Likewise, a study on attitudes and preferences of surgical oncology fellows on preparing for their certification process supported the same preferences. This study noted that fellows were unsure of how to prepare and that added to their anxiety about their exam [16]. Establishing a longitudinal mentorship program related to board review and preparation could be one way for programs to address these preferences and assist residents in succeeding with preparation efforts. A formal, peer-led board preparation mentorship program could be valuable for trainees since peer mentors could also provide valuable psychosocial support in addition to advice on study methods [18]. One of the specific recommendations from our residents was a preference for templates for studying or having a centralized list of board preparation resources and methods.

Residents also reported favoring more organized and integrated focus on board preparation from the residency program, and they desired greater accountability from the program for their study efforts. Accountability, with consequences and incentives, was shown to be moderately effective in increasing psychiatry resident in-training scores [19]. In this study accountability measures included requirements such as mandatory meetings with a mentor who gave practice questions for structured study hall time and loss of moonlighting privileges if certain testing scores were not met. Such specific requirements, when also supported by the residents and not felt to be completely external in nature, could help residents prepare and know that they are spending significant time preparing.

Participants across all training years reported that preferred board preparation methods were likely to vary among residents, relating a variety of preferences themselves and indicating that this was representative of the larger group of trainees. While participants particularly focused on studying board style questions as a preferred method to prepare for boards, which matches the predominant preference found in other studies, there were a variety of additional preferred learning methods identified by other individuals in the groups [17]. Residents stated that timing and frequency of learning was important, with participants preferring paced learning and incorporation of efforts early in training. A study on a text-based question and answer exam preparation platform showed that one of the positives features according to participants was that the program gave daily content and employed a rich question/answer format [9]. Residency programs could consider implementing a system of consistent question delivery, such as a ‘question of the day’ for residents. Group work was another preference identified by some participants. A study of team-based learning (TBL) feasibility in an internal medicine residency program showed that the residents thought they learned more medical knowledge through this approach than through lectures [20].

Our residents also expressed interest in interactive learning formats and competitive learning methods. Research in gamification within medical education aligns with this point. Recently, a report of internal medicine residents, who were surveyed after using a team or individual competition based on answering questions correctly, noted the most important motivator for their participation was the use of a low stakes leaderboard [8]. In one intervention, answering weekly questions delivered through email was correlated with better ABP CE performance results [3]. Similarly, a texting program which delivered a combination of commonly tested facts and questions to pediatrics residents was associated with improvements in their In-Training Exam (ITE) results [9]. Another residency program used a board review course method for pediatric residents who were at risk of failing the boards and described positive results with this intervention [21]. In family medicine residents, online module completion was associated with passing the certification exam and better scores [4]. Spaced, test enhanced learning has also been shown to improve medical knowledge retention in medical students and residents [22, 23]. Importantly, in a study surveying the study habits of anesthesiology residents, investigators found that the specific types of study methods did not correlate with the ITE scores of residents, and interestingly, residents believed that program support for their study effort, whichever they chose, was important to them [7]. This literature, along with the comments and preferences of our residents, underscores the value of organized programmatic efforts to promote board examination preparation, the willingness of trainees to participate, and the effectiveness of a variety of defined approaches. Table 3 describes potential interventions to address each of the themes identified by the pediatric residents.

**Table 3:** Potential Interventions to Improve Board Preparation Linked to Themes Identified by Pediatric Residents

Theme	Potential Interventions
Not of Immediate Importance	Begin addressing board preparation early in first year; Require specific board preparation activities in first year (e.g., board preparation questions, board preparation study materials, etc.) and continue through program.
Personalized Guidance	Provide individualized discussion and board preparation ‘prescription’ for each resident; Provide list of endorsed board preparation and board study resources to assist residents; Develop program of peer, senior resident or graduate advisors/mentors for board preparation; For residents struggling on ITE scores, provide consultation counselor experienced in studying methods and skills to provide personalized testing and advice.
Increased focus from the program	Implement requirements for board preparation and link to external rewards (e.g., academic funds, moonlighting permission, etc.); Link review of ITE scores with defining expectations of board preparation goals for each resident; Incorporate board preparation activities into regular resident didactic sessions; Implement board review series/course for residents; Implement a ‘board question of the day’ program for residents; Explore study hall scheduling, potentially for residents with poor ITE scores.
Individualized Learning Format	Provide wide spectrum of endorsed board preparation materials and methods for residents (e.g., question banks, videos, podcasts, study/reading materials, etc.); Provide funding for residents to purchase board preparation materials specifically chosen by the resident.

While these results can help inform pediatric residency programs on resident preferences for board preparation there are limitations to our study. Focus groups were conducted at a single center with a limited sample size and may not be reflective of all perspectives. Each focus group represented only a fraction of the pediatric residents in our institution. The focus group coding methodology may have introduced bias as the authors of the study coded and performed the thematic analysis of the study. There was, however, good consensus between the four coders who evaluated the transcripts.

## Conclusions

We found that residents 1) struggle to prepare since they find the certification exam, given after graduation from residency, not of immediate importance; 2) would like more guidance from the program regarding board preparation materials and methods; 3) welcome more emphasis and accountability placed on them regarding board preparation; and 4) desire a variety of learning formats to meet the different preferences of different residents. Further work in this area could include a multi-center exploration of the validity of these themes and more efforts to match certifying examination preparation methods to resident preferences to determine if these efforts augment resident performance on this examination.
